# Cutaneous Pyogranulomas Associated with *Nocardia* *jiangxiensis* in a Cat from the Eastern Caribbean

**DOI:** 10.3390/tropicalmed4040130

**Published:** 2019-10-17

**Authors:** Adam Silkworth, Ryan Cavanaugh, Pompei Bolfa, Anne A.M.J. Becker

**Affiliations:** 1 Department of Clinical Sciences, Ross University School of Veterinary Medicine, St. Kitts KN0602, West Indies; RCavanaugh@rossvet.edu.kn; 2 Ross University Veterinary Clinic (RUVC), Ross University School of Veterinary Medicine, St. Kitts KN0602, West Indies; 3 Department of Biomedical Sciences, Ross University School of Veterinary Medicine, St. Kitts KN0602, West Indies; PBolfa@rossvet.edu.kn (P.B.);

**Keywords:** nocardiosis, feline, skin and subcutis, actinomycetoma, Caribbean, One Health

## Abstract

*Nocardia* spp. are worldwide, ubiquitous zoonotic bacteria that have the ability to infect humans as well as domestic animals. Herein, we present a case of a five-year-old female spayed domestic shorthair cat (from the island of Nevis) with a history of a traumatic skin wound on the ventral abdomen approximately two years prior to presenting to the Ross University Veterinary Clinic. The cat presented with severe dermatitis and cellulitis on the ventral caudal abdomen, with multiple draining tracts and sinuses exuding purulent material. Initial bacterial culture yielded *Corynebacterum* spp. The patient was treated symptomatically with antibiotics for 8 weeks. The cat re-presented 8 weeks after the initial visit with worsening of the abdominal lesions. Surgical intervention occurred at that time, and histopathology and tissue cultures confirmed the presence of *Nocardia* spp.-induced pyogranulomatous panniculitis, dermatitis, and cellulitis. Pre-operatively, the patient was found to be feline immunodeficiency virus (FIV)-positive. The patient was administered trimethoprim/sulfamethoxazole (TMS) after antimicrobial sensitivity testing. PCR amplification and 16S rRNA gene sequencing confirmed *Nocardia* *jiangxiensis* as the causative agent. To our knowledge, *N. jiangxiensis* has not been previously associated with disease. This case report aims to highlight the importance of a much-needed One Health approach using advancements in technology to better understand the zoonotic potential of *Nocardia* spp. worldwide.

## 1. Introduction

Nocardiosis is a potentially zoonotic disease affecting both humans and animals worldwide. *Nocardia* spp. are aerobic Gram-positive bacteria responsible for causing localized or disseminated infections in animals and humans. The bacteria are most commonly found in soil, organic material, fresh and saltwater, dust, compost vegetation, as well as other environmental sources [1,2]. The virulence and pathogenicity of *Nocardia* spp. is attributable to the mycolic acids in the cell wall and the ability to resist the neutrophil and activated macrophage attack (cell-mediated immune response) by inhibiting phagosome–lysosome fusion in these cells [2]. Nocardiosis is a rare, albeit serious, infectious disease and develops when the ubiquitous soil saprophyte is inadvertently inoculated into skin puncture wounds or inhaled into the lungs [3]. In humans and animals, *Nocardia* spp. may manifest in the pulmonary form, cause cutaneous lesions, or induce systemic disease in the disseminated form. The most common presentation in domestic animals is cutaneous, whereas the most common presentation in humans tends to be the pulmonary form; however, all forms have been reported in both animals and humans [1,4]. Transfer of infection between animals or transmission from diseased animals to humans has only been demonstrated in recent years, with the incidence and prevalence of potential zoonosis still unknown. However, nocardiosis has been reported in humans with profound scratch or bite wounds from clinically healthy cats or dogs [5]. *Nocardia* is labelled as an opportunistic pathogen, with the majority of cases occurring in immunocompromised hosts; however, documentation of the disease in immunocompetent individuals has been sporadically confirmed [6].

The veterinarian Edmond Nocard, who isolated a Gram-positive organism thought to be the causative agent in a case of bovine farcy, reported the first documented case of *Nocardia* in an animal in 1888 on the island of Guadeloupe in the West Indies. This strain was given the name *Nocardia farcinica* in 1889 by Trevisan, who defined the genus that included Nocard’s *N. farcinica* isolate and five other similar species [7]. The first human case of nocardiosis (caused by *Nocardia asteroides*) was reported by Eppinger in 1890 [6]. Eppinger isolated a similar aerobic, branching filamentous organism to that of Nocard from a human with a fatal brain abscess and called this organism *Cladothrix asteroides*. Six years later, in 1896, it was renamed *Nocardia asteroides* [4,7]. 

We describe a case of an immunocompromised cat infected with the cutaneous form of *Nocardia jiangxiensis* on the Caribbean island of Nevis, in the Federation of St. Kitts and Nevis, and aim to highlight the importance of a One Health approach and the need to better understand and classify *Nocardia* spp. across all domestic animals and humans alike. Considering nocardiosis as a worldwide disease with reported zoonotic events, the integration of data and outcomes acquired across all species is essential to advance our knowledge of this disease, as a whole, in the medical profession. This is the first known identification of *N. jiangxiensis* associated with clinical disease in a human or animal species, to the author’s knowledge. 

## 2. Case Presentation 

**Figure 1:**
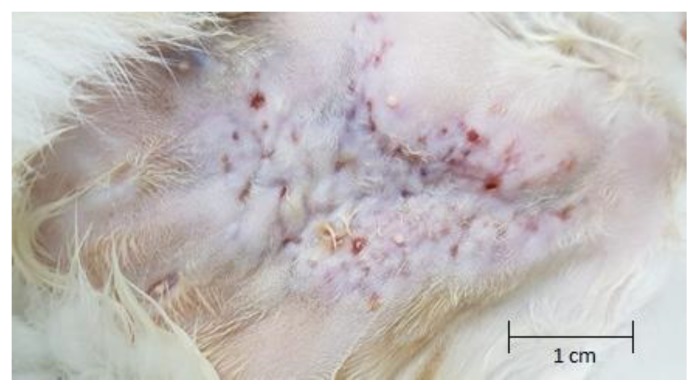
The skin on the caudal ventral abdomen on original presentation. Note no hair had been shaved here.

A five-year-old, 4 kg, female spayed domestic shorthair cat, originally from the island of Sint Eustatius, presented to the Ross University Veterinary Clinic (RUVC) from the island of Nevis, in the federation of St. Kitts and Nevis, for a chief complaint of non-healing dermal lesions located on the ventral abdomen. Mild pyrexia was confirmed (rectal temperature 39.5 °C [normal range 37–39 °C]); however, other vital parameters were within normal limits. Physical examination revealed a 4 cm × 5 cm region of alopecia centered over the caudal aspect of the ventral abdomen. The area of affected dermis was thickened with numerous punctate full-thickness wounds, which were determined to be draining fistulous tracts, as shown in Figure 1. Purulent discharge was exuding from a number of the tracts. An aerobic culture and sensitivity test was carried out at that time, which yielded few (2+) *Corynebacterium* spp. The cat was administered a subcutaneous injection of a long-acting (2 weeks) cephalosporin and one dose of a non-steroidal anti-inflammatory (NSAID)—robenacoxib—subcutaneously. When the cat returned 2 weeks later for reevaluation, the dermal lesions appeared unchanged, and an additional injection of cefovecin was administered. The sensitivity results had not returned at that time. Once returned, it was reported that no Clinical Laboratory Sciences Institute (CLSI) interpretation was available for the cefovecin. 

The owner contacted the RUVC two weeks later to report that the cat’s dermal lesions were progressing despite the prescribed therapy. The sensitivity results showed the *Corynebacterium* spp. to be susceptible to amoxicillin/clavulanic acid. The patient was prescribed amoxicillin/clavulanic acid twice daily by mouth. The owner contacted the RUVC 19 days later to report that there was no improvement in the cat’s condition. A recheck appointment with possible surgical intervention was requested at that time, to which the owner obliged. 

The cat presented to the RUVC for reevaluation 8 weeks after initial presentation. The fistulous tracts and area of pathology appeared significantly worse than on original presentation. The decision was made for radical en bloc resection with tissue cultures and biopsy. Pre-operative blood work was performed, and the cat was confirmed to be FIV (feline immunodeficiency virus)-positive using the IDEXX Snap feline leukemia virus (FeLV)/FIV combination test. A complete blood count (CBC) showed a very mild hypoproteinemia of 5.7 g/dL (reference range (RR) 6.0–7.5 g/dL), and serum biochemistry revealed hypercalcemia at 13.1 mg/dL (RR 8.0–11.8 mg/dL) and decreased alkaline phosphatase (ALP) of 0 U/L (RR 10–90 U/L). No other abnormalities were noted, and the cat was deemed an appropriate candidate for surgery. 

En bloc resection of all grossly abnormal abdominal tissue was performed. This included skin, subcutaneous tissue, and caudal mammary glands (3rd and 4th) bilaterally, as well as an 8 cm (length) × 4 cm (width) section of external rectus sheath or body wall. An attempt was made to include a 1 cm lateral margin of macroscopically normal tissue, while leaving enough tissue to facilitate reconstruction of the wound. The cat recovered without incident from surgery and anesthesia. The cat was hospitalized for 48 h post-operatively without complications. She was discharged to the owner, sent home on pain management, and placed on enrofloxacin every 12 h for 14 days, while awaiting the histopathology and culture and sensitivity results. 

**Figure 2:**
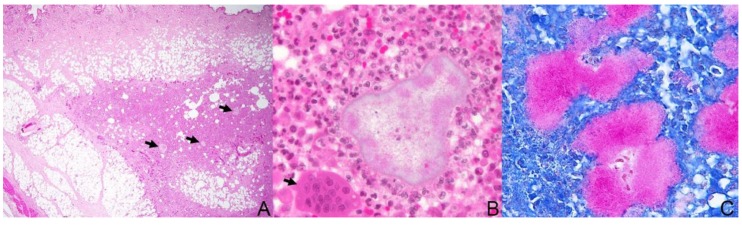
Haired skin, cat. (**A**) Nodular pyogranulomatous panniculitis with bacterial colonies (black arrows, actinomycetoma); hematoxylin and eosin (HE) staining. (**B**) Detail of Figure 2A showing a large, irregular, pale basophilic filamentous bacterial colony (center) surrounded by many neutrophils, fewer macrophages, and a multinucleated giant cell (arrow); HE staining. (**C**) The filamentous bacilli were partially acid-fast, suggestive of *Nocardia* spp.; Fite staining.

All excised tissue was submitted for histopathologic evaluation, as well as aerobic and anaerobic culture and sensitivity testing. The formalin-fixed tissue was processed routinely, sectioned at 5 µm, and stained with hematoxylin and eosin (HE). Additionally, special stains were performed after the initial HE assessment: Gram (Brown and Brenn and Brown-Hopps) and Coates modified Fite stain (a modification of the Ziehl–Neelsen technique). Most of the slides examined showed within the subcutis and sometimes extending into the panniculus, carnosus and/or adipose tissue multifocal to coalescing nodular aggregates, predominantly with neutrophils (viable and degenerate) and smaller amounts of macrophages, some epithelioid and multinucleated macrophages, and fewer lymphocytes and plasma cells, admixed with areas of necrosis, fibrin, and hemorrhage (Figure 2A). Some areas of pyogranulomatous inflammation were surrounded by fibrosis, and in some areas, epithelial ulceration and fistulous tracts were seen connecting the deeper lesions with the skin surface. Randomly scattered through these areas were large, pale, poorly stained, basophilic filamentous bacterial colonies, surrounded by a clubbed fine corona of eosinophilic Splendore–Hoeppli material (‘sulfur granules”) (Figure 2B). The bacterial colonies were Gram-positive and stained stronger with the Fite modified acid-fast stain (Figure 2C). The morphological diagnosis for the lesion was multifocal nodular pyogranulomatous panniculitis, dermatitis, and cellulitis with filamentous bacterial colonies suggestive of *Nocardia* spp. (actinomycetoma).

Communication from the owner of the cat 3 weeks after surgery confirmed that there were de novo lesions appearing around the surgical scar on the ventral abdomen. The patient was prescribed liquid trimethoprim/sulfamethoxazole (TMS) on the suspicion of *Nocardia* spp. from the histopathologic findings, while awaiting the final culture and sensitivity results.

The aerobic bacterial cultures yielded a Gram-positive filamentous rod that was catalase-positive, acid-fast-negative, and indole-negative. Anaerobic culture yielded no growth. Fungal culture yielded no fungal growth, but the plate became overgrown with bacteria. With the negative anaerobic culture and the positive aerobic culture results in combination with the histopathologic findings of partially acid-fast (Fite stain) filamentous bacilli, we were able to identify *Nocardia* spp. as the inciting cause of the dermal lesions in this case. The final culture and sensitivity results confirmed *Nocardia* spp. and showed susceptibility to TMS, which the cat had already been prescribed. A CBC was performed 4 weeks after beginning the antibiotic treatment, and no abnormalities were noted. 

**Figure 3:**
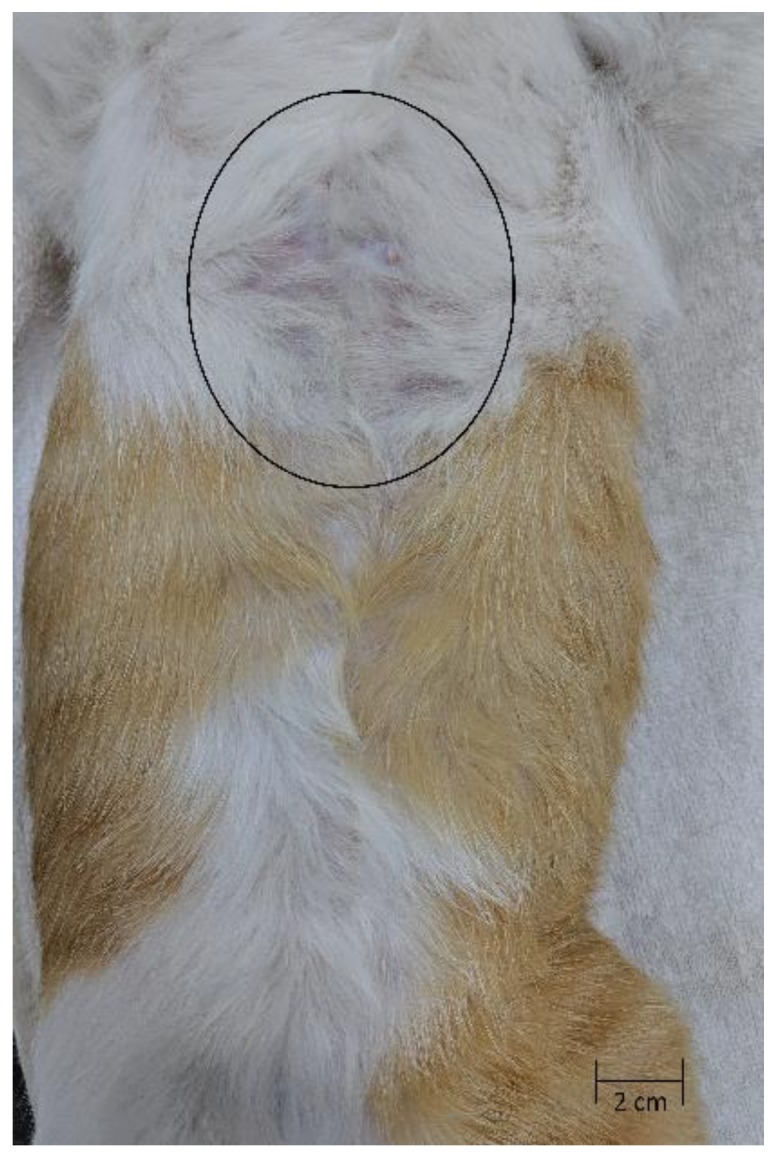
Seven weeks after TMS initiation (11 weeks post-operatively). A mild discoloration of the skin (black circle) was noted on physical exam. Note the hair regrowth over the entire abdomen.

The cat presented for a recheck 7 weeks after the institution of the TMS therapy (Figure 3). The cat was clinically well, and the owner noted a significant improvement in her attitude and eating habits. There were no dermal lesions evident at that time; however, a mild discoloration of the skin at the cranial-most portion of the surgical site was noted. The cat was continued on the TMS treatment for an additional 5 weeks (12 weeks total), then the medication was discontinued. 

The cat had been off medication for 11 days when the owner reached out with concerns that a single de novo lesion was observable on the caudal abdomen. Physical exam upon recheck revealed a 5 cm × 5 cm well-circumscribed circular, moderately firm subcutaneous mass with a single, 1 mm red lesion in the dermis. The TMS therapy was immediately reinstituted, and the cat was still being treated at the time this case report was submitted for publication.

Upon further evaluation of the *Nocardia* spp. isolates obtained from the tissue culture samples, *N. jiangxiensis* was identified in this case using PCR amplification and 16S rRNA gene sequencing. Upon initial evaluation of approximately 500 base pairs of 16S rRNA gene sequencing covering the V3–V4 region and using universal primers (UVBF and UVBR), nucleotide analysis of isolate #1 (ZV7487) revealed 97.4% (366/376) homology with *N. jiangxiensis* strain 43401^T^ and 96.81% (364/376) homology with *Nocardia rayongensis* strain RY45-3 and with *Nocardia nova* strain ATCC 33726 using BLAST search [8]. A partial analysis performed on isolate #2 (ZV7488) revealed only 92.24% (297/322) homology with *N. jiangxiensis* strain 43401^T^ and 91.61% (295/322) homology with *N. rayongensis* strain RY45-3 and with *N. nova* strain ATCC 33726. This is the first clinical case of *N. jiangxiensis* reported in an animal or human, to the author’s knowledge.

## 3. Discussion

The identification and classification of *Nocardia* has changed drastically over the years. With many similarities among different strains of *Nocardia* and the taxonomic transition, identification to the species level has become important. With advances in molecular genetics, there have been significant improvements in the ability to identify the different *Nocardia* spp., thus moving away from phenotypic identification. To date, more than 90 species of *Nocardia* are recognized worldwide. Over 30 species have been described as causing opportunistic infections in humans, and at least 30 species have been deemed responsible for animal diseases, with immunocompromised host being at a greater risk of contracting the disease [2]. There have recently been large taxonomic changes in *Nocardia* classification due in part to the utilization of ever-developing methods of molecular classification techniques, and new species are being described and identified at a rapid pace [2]. The List of Prokaryotic Names with Standing in Nomenclature (LPSN, http://www.bacterio.net/nocardia.html) currently holds 92 globally recognized species of *Nocardia* with valid names. Of these 92, there are 54 species that have been shown to be clinically significant on the basis of having at least one published peer-reviewed report [7]. 

To our knowledge, this is the first report associating *N. jiangxiensis* with clinical disease in an animal, adding this species of *Nocardia* to the list of nocardial pathogens. *N. jiangxiensis* has been a documented species since 2005, when it was first discovered and isolated from rhizosphere soil of goose grass next to a copper mine in Wushan, Jiangxi Province, in southern China [9]. Upon its discovery, *N. jiangxiensis* was found to have to 98.7% and 97.9% 16S rRNA gene sequence similarity with *N. nova* JCM 6044^T^ and *Nocardia pseudobrasiliensis* ATCC 51512^T^, respectively. This isolate had DNA–DNA relatedness values and corresponding relatedness values of 46% with *N. nova* JCM 6044^T^ and of 39% with *N. pseudobrasiliensis* ATCC 51512^T^, values that are well below the recommended threshold of 70% for genomic species differentiation, and was thus deemed a novel species after full analysis (GenBank Accession number AY639902) [9]. The cat in this case had no travel history outside of the West Indies island chain in the Caribbean, so it is hypothesized that this species exists well outside southern China, and its global reach is yet to be determined. This highlights the importance of molecular species-level identification, as certain species are yet to be considered infectious but have not been properly identified until recent years. 

*Nocardia* spp. vary by geographic region. They may present with different pathogenic traits and have even shown differences in antimicrobial susceptibility patterns. This makes the differentiation and identification of *Nocardia* isolates at the species level important so as to provide the appropriate level of patient care, whether animal or human [7]. Two genetic methods have historically been used in the identification of *Nocardia* at the species level, i.e., PCR–restriction enzyme pattern analysis (PRA) of the *hsp65* gene and the 16S rRNA gene sequencing method. The PRA-*hsp65* gene analysis method has been evaluated as not as discriminatory at the species level, although still beneficial, compared to the 16S rRNA gene sequencing method, and it has been proposed that combining the sequences from these two types of analysis could form the basis for a new *Nocardia* species identification system, particularly for species with similar 16S rRNA sequences [10]. Further development of a potential new system, by combining these two methods, would serve to benefit veterinary and human medicine alike and could lead us closer to a One Health approach. 

Typical nocardial infections in domestic animals produce suppurative to pyogranulomatous inflammation that manifests as one of three typical clinical presentations: cutaneous, pulmonary, and systemic or disseminated [1,4,11,12]. Cutaneous nocardiosis is the most common presentation in cats and usually involves the extremities, inguinal area (as in this case), and neck. Morphologically, cutaneous lesions caused by *Nocardia* spp. may present as abscesses, cellulitis, and fistulous draining tracts [10,11,12,13]. The majority of reported cases in animals are caused by species of the *N. asteroides* complex; however, other common species known to cause infection include *Nocardia brasiliensis, N. pseudobrasiliensis, N. nova*, and *Nocardia otitidiscaviarum* [14,15]. *N. brasiliensis* is the most often reported cause of cutaneous and lymphocutaneous disease in people and is more common in tropical regions, whereas *N. asteroides* complex is responsible for approximately 80% of systemic or disseminated nocardiosis cases in humans and accounts for a certain number of central nervous system infections worldwide [1,4].

Cutaneous nocardiosis may initially start as an abscess; however, it frequently develops into an exudative non-healing wound that may become suppurative in certain cases. This can sometimes occur following preliminary surgical intervention without adequate removal of the lesion or initiation of inappropriate antimicrobial therapy [1]. Surgical intervention with adequate removal of the diseased tissue, along with proper antimicrobial therapy, led to the eventual resolution in our case. However, remission did not occur initially, as the antimicrobial sensitivity of the surgically procured culture showed resistance to enrofloxacin and a susceptibility to trimethoprim/sulfamethoxazole, and only after the sulfonamide antibiotic was initiated, did we see definitive resolution of the clinical signs. 

Initial *Nocardia* lesions tend to radiate outwardly, with subsequent development of encompassing contiguous lesions in which draining sinus tracts often ensue [1]. The cat in this case suffered a traumatic penetrating injury on its caudal abdomen 2 years prior to this hospital visit, after jumping over a metal fence and injuring itself. The wound occurred years after moving from the island of Sint Eustatius, so it can be assumed with confidence that the infection was acquired in Nevis, as the cat had no other travel history. The injury appeared initially to heal on its own without medical intervention; however, the wound would open and heal periodically according to the owner. We assume that the original injury subsequently developed into the progressive radiating lesions observed on initial presentation, but it is impossible to discern if the inoculation of *N. jiangxiensis* took place at the time of the original injury or during an episode of the periodic wound dehiscence.

Reports on clinical experience in human patients have shown that a successful resolution of clinical signs may require the use of proper antimicrobial therapy combined with surgical intervention, which was extrapolated in this case. There have been reported cases of feline mycobacterial panniculitis that have benefitted from en bloc resection and wound reconstruction, an approach that has historically proved effective and thus should always be considered in nocardial infections in animals [1,16,17]. The need for identification of the inciting cause with tissue cultures and histopathologic exam, along with the worsening symptoms after 8 weeks of treatment, all contributed to the decision of surgical debridement and outweighed the potential risks of waiting and continuing to treat the cat empirically. Para-aminobenzoic acid is a component of *Nocardia* exudates, and its formation is the rate-limiting step in substrate inhibition by sulphonamide drugs, which makes the removal of purulent material an essential component of therapy in cutaneous nocardial infections [1]. Patients with thick-walled abscesses or mycetomas may require aggressive surgical debridement and drainage to achieve full resolution, as was the case here [1]. Because of the extent, severity, and progression of the dermal lesions in this case, both surgical intervention and proper antimicrobial therapy were essential to achieve resolution of the lesion. This case highlights the challenges in the diagnosis of nocardiosis and the call for both surgical and antimicrobial therapies to be considered together as the standard of care for *Nocardia* treatment, for humans and animals alike.

## 4. Conclusions

We demonstrated the presence of *N. jiangxiensis* as a cause of cutaneous pyogranulomas in a cat in the federation of St. Kitts and Nevis, in the West Indies Caribbean island chain. Although no zoonotic transmission was reported in this case, the discovery of a new species that infects animals highlights the need for a One Health approach to deal with this worldwide ubiquitous organism. The emergence of a new infective species in animals demonstrates the need that all confirmed *Nocardia* cases, whether animal or human, be identified at the species level and integrated across all medical fields to better understand the geographic reach, prevalence, and outcomes of this disease.

## Ethics Statement

The Ross University School of Veterinary Medicine Institutional Animal Care and Use Committee (IACUC) was informed of the tissues that were surgically procured and found this case report exempt from their standards, as this was a client-owned animal (IACUC #TSU9.2.19). We received informed owner consent to compose this case report and demonstrated best practices in veterinary surgery and medicine in the diagnosis and treatment of this subject.
